# Correction: Sung et al. Linear Tumor Regression of Rectal Cancer in Daily MRI during Preoperative Chemoradiotherapy: An Insight of Tumor Regression Velocity for Personalized Cancer Therapy. *Cancers* 2022, *14*, 3749

**DOI:** 10.3390/cancers16122220

**Published:** 2024-06-14

**Authors:** Soo-Yoon Sung, Sea-Won Lee, Ji Hyung Hong, Hye Jin Kang, So Jung Lee, Myungsoo Kim, Ji-Hoon Kim, Yoo-Kang Kwak

**Affiliations:** 1 Department of Radiation Oncology, Eunpyeong St. Mary’s Hospital, Catholic University of Korea College of Medicine, Seoul 06591, Republic of Korea; ssy-uni@hanmail.net (S.-Y.S.); lords_seawon@hotmail.com (S.-W.L.); 2 Division of Medical Oncology, Department of Internal Medicine, Eunpyeong St. Mary’s Hospital, Catholic University of Korea College of Medicine, Seoul 06591, Republic of Korea; jh_hong@catholic.ac.kr; 3 Department of Radiation Oncology, Incheon St. Mary’s Hospital, Catholic University of Korea College of Medicine, Seoul 06591, Republic of Korea; jade2959@hanmail.net (H.J.K.); sunport@naver.com (S.J.L.); mskim0710@gmail.com (M.K.); 4 Department of Surgery, Incheon St. Mary’s Hospital, The Catholic University of Korea, Seoul 06591, Republic of Korea; samryong@catholic.ac.kr

In the original publication [1], support from Clinical Trials Center of Incheon St. Mary’s Hospital, The Catholic University of Korea, was not included. The authors apologize for any inconvenience caused and state that the scientific conclusions are unaffected. This correction was approved by the Academic Editor. The original publication has also been updated.
