# Supplementary material for: Linear Tumor Regression of Rectal Cancer in Daily MRI during Preoperative Chemoradiotherapy: An Insight of Tumor Regression Velocity for Personalized Cancer Therapy
Source: Cancers (Basel). 2022 Aug 1;14(15):3749. doi: 10.3390/cancers14153749 (PMC9367607; doi:10.3390/cancers14153749)
Supplement: Supplementary file 1 [file cancers-14-03749-s001.zip › cancers-1815811-supplementary.pdf]

cancers

MDPI

Supplementary Material: Linear Tumor Regression of Rectal Cancer in Daily MRI during Pre-operative Chemoradiotherapy: An Insight of Tumor Regression Velocity for Personalized Cancer Therapy

Soo-Yoon Sung, Sea-Won Lee, Ji Hyung Hong, Hye Jin Kang, So Jung Lee, Myungsoo Kim, Ji-Hoon Kim and Yoo-Kang Kwak

Table S1. Daily tumor volume of 20 patients who underwent chemoradiotherapy showed significant regression from baseline as early as fourth fraction.

**Tumor volume (cc)**										
	**Fx 1**	**Fx 2**	**Fx 3**	**Fx 4**	**Fx 5**	**Fx 6**	**Fx 7**	**Fx 8**	**Fx 9**	**Fx 10**
Mean	81.28	76.04	70.96	67.33	67.13	64.39	61.02	58.19	57.02	53.30
SD	48.77	48.17	42.31	40.55	40.81	41.66	37.00	35.31	33.41	29.27
Median	91.30	83.34	78.17	75.63	74.95	72.68	72.02	67.55	66.42	63.09
Min	8.77	8.65	7.96	5.73	7.32	7.08	7.61	7.36	8.48	7.45
Max	344.51	305.43	297.72	288.81	291.47	288.06	274.78	254.34	243.09	227.45
P	-	0.100	0.003	< 0.0001	< 0.0001	< 0.0001	< 0.0001	< 0.0001	< 0.0001	< 0.0001
	**Fx 11**	**Fx 12**	**Fx 13**	**Fx 14**	**Fx 15**	**Fx 16**	**Fx 17**	**Fx 18**	**Fx 19**	**Fx 20**
Mean	43.12	42.25	33.10	39.07	37.13	35.60	34.86	28.31	31.62	24.26
SD	22.06	20.73	18.31	18.81	19.65	17.62	15.43	13.53	14.60	11.98
Median	53.93	52.70	44.61	51.65	48.55	46.45	44.46	38.62	38.72	29.55
Min	7.31	7.53	5.51	4.65	4.64	3.87	5.21	5.38	3.60	3.64
Max	218.10	209.25	206.19	207.22	194.96	183.91	175.85	175.46	142.76	131.36
P	< 0.0001	< 0.0001	< 0.0001	< 0.0001	< 0.0001	< 0.0001	< 0.0001	< 0.0001	< 0.0001	< 0.0001

cancers MDPI

	Fx 21	Fx 22	Fx 23	Fx 24	Fx 25
Mean	29.34	29.39	26.04	24.50	23.04
SD	12.34	12.60	12.47	12.47	10.79
Median	36.61	36.75	33.33	31.20	29.04
Min	3.71	3.69	2.66	2.90	1.50
Max	129.85	132.21	127.30	124.56	115.14
P	< 0.0001	< 0.0001	< 0.0001	< 0.0001	< 0.0001

Abbreviations: Fx = fraction, SD = standard deviation, min = minimum, max = maximum

cancers

MDPI

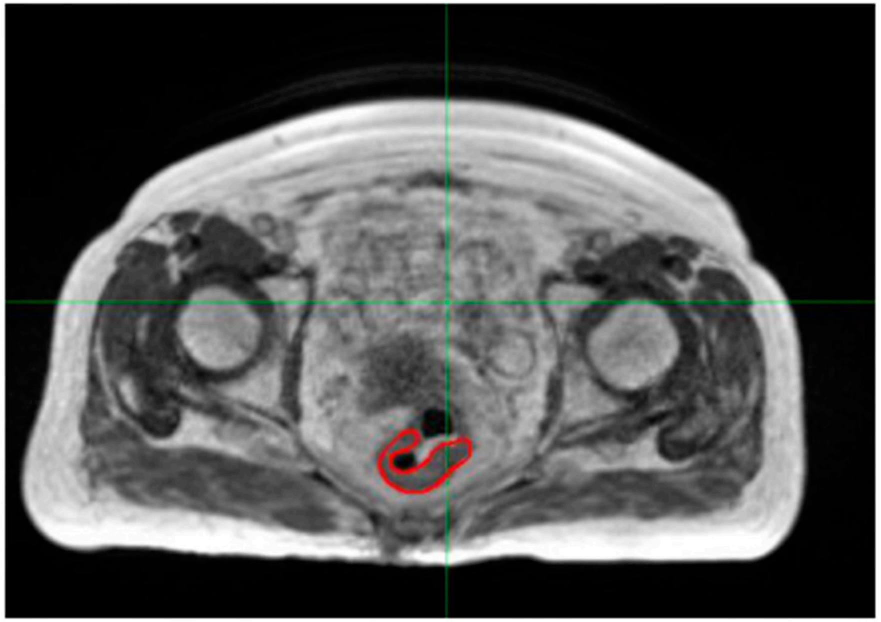

Figure S1. An example of contouring tumor volume in an axial slice of registration image
